# Hemodynamic Monitoring by Smartphone—Preliminary Report from a Comparative Prospective Observational Study

**DOI:** 10.3390/jpm12020200

**Published:** 2022-02-01

**Authors:** Michał P. Pluta, Magdalena Dziech, Mateusz N. Zachura, Anna J. Szczepańska, Piotr F. Czempik, Piotr S. Liberski, Łukasz J. Krzych

**Affiliations:** 1 Department of Anaesthesiology and Intensive Care, Faculty of Medical Sciences in Katowice, Medical University of Silesia, Medyków 14 Street, 40752 Katowice, Poland; aszczepanska@sum.edu.pl (A.J.S.); pczempik@sum.edu.pl (P.F.C.); pliberski@sum.edu.pl (P.S.L.); lkrzych@sum.edu.pl (Ł.J.K.); 2 Emergency Medicine Department, St. Barbara’s Memorial Hospital No. 5 Trauma Center, Medyków 1 Square, 41200 Sosnowiec, Poland; 3 Students’ Scientific Society, Department of Anaesthesiology and Intensive Care, Faculty of Medical Sciences in Katowice, Medical University of Silesia, 40752 Katowice, Poland; magdalena.dziech@gmail.com (M.D.); mateusz.zachura@gmail.com (M.N.Z.)

**Keywords:** cardiac output, hemodynamic monitoring

## Abstract

Background: Advanced hemodynamic monitoring supports making therapeutic decisions in critically ill patients. New technologies, including mobile health, have been introduced into the hemodynamic monitoring armamentarium. However, each monitoring method has potential limitations—content, technical and organizational. The aim of this study was to assess the comparability between measurements obtained with two arterial pressure cardiac output methods: Capstesia™ smartphone hemodynamic software (CS) and LiDCO Rapid™ uncalibrated hemodynamic monitor (LR). Methods: The initial analysis included 16 patients in the period 06–09 2020 without limitations that could make the results obtained unreliable. Eighty pairs of cardiac output measurements were obtained. The comparability of cardiac output results obtained with both methods was assessed using the Spearman’s rank correlation coefficient (R), the intra-class correlation (CCC) and the Bland–Altman curves analysis (B-A). Results: The median (IQR) cardiac output measured with CS and LR were 4.6 (3.9–5.7) and 5.5 (4.6–7.4) L min^−1^, respectively. In the B-A analysis, CS cardiac output values were on average 1.2 (95% CI −2.1–4.4) L min^-1^ lower than LR values. The correlation between cardiac output with CS and LR was moderate (*r* = 0.5; *p* = 0.04). After adjusting for the presence of the dicrotic notch on the pulse waveform, in the group of eight patients with a visible dicrotic notch, the CS and LR results differed by only 0.1 (95% CI −0.8–1.1) L min^−1^, the correlation between CS and LR was close to complete (*r* = 0.96; *p* < 0.001), and the percentage error was 40%, with a CCC-CS of 0.98 (95% CI 0.95–0.99). Conclusions: The Capstesia^TM^ smartphone software can provide an alternative method of cardiac output assessment in patients meeting arterial pressure cardiac output evaluation criteria with a clearly discernible dicrotic notch on the arterial pulse pressure waveform. It is necessary to confirm the obtained observations on a larger group of patients; however, it may potentially make objective hemodynamic measurements ubiquitous in patients with invasive arterial pressure monitoring with a clearly discernible dicrotic notch.

## 1. Introduction

Restoring and maintaining cardiovascular stability is one of the basic tasks of critical care physicians. Therapy with fluids and inotropic and vasoactive drugs is aimed at obtaining the cardiac output (CO) and systemic vascular resistance (SVR) adequate to patient needs, ultimately ensuring optimal organ perfusion [1]. Due to the complex pathophysiology of circulatory disorders, hemodynamic management should be based on parameters derived from hemodynamic monitors [2].

In the initial phase of management of critically ill patients, which is often carried out in an emergency department (ED), there is usually no technical or organizational capacity to implement complex hemodynamic monitoring (e.g., transpulmonary thermodilution). Advanced hemodynamic monitoring is also rarely used in the operating room [3]. However, arterial cannulation is relatively safe and fast, which makes it possible to assess CO in selected patients based on the analysis of the arterial-pressure-based cardiac output (APCO) curve [4]. The end of the period of contraction on this line can be seen as a dicrotic notch, which is located on the descending arm and corresponds to the closure of the aortic valve [5]. The stroke volume is proportional to the area under this curve in the systolic phase.

Repeated CO assessment allows for individualized, goal-directed therapy by identifying the pathomechanism of circulatory disorders, optimizing the pharmacological support of the circulatory system, and evaluating the response to the applied therapy in real time.

The usefulness of mobile applications (m-health) in patient care has been the subject of many studies. Desebbe et al. demonstrated the usefulness of a smartphone for non-invasive BP measurement in non-cardiac surgery patients hospitalized in a postoperative care unit [6]. Ultrasound diagnostics based on portable ultrasound heads connected with a smartphone are the basic diagnostic tool at the patient’s bedside [7].

Until recently, the limitation on the widespread use of APCO was the need for costly stand-alone devices operating on the basis of the manufacturer’s proprietary algorithms. Like other applications used in medicine [8,9], the launch of the more affordable Capstesia™ (Galenic App, Vitoria-Gasteiz, Spain) (CS) potentially relegated the smartphone to the role of an advanced hemodynamic monitor, accessible to all practitioners, regardless of where services were provided [10].

The studies that have been conducted so far are promising, but they include patients anesthetized in the operating room who are monitored by methods other than LidcoRapid™ technology (Lidco, Cambridge, UK) (LR) [11,12,13]. The aim of our study was to analyze the comparability of CO measurements obtained with the CS application and the uncalibrated LR monitor in patients hospitalized in the intensive care unit (ICU). To the best of our knowledge, no comparison of the two devices has been made before. Additionally, both devices do not require calibration, which eliminates the influence of potential disturbing factors on the obtained results.

## 2. Materials and Methods

We conducted a prospective, observational, comparative study of two methods of CO monitoring. The population studied included patients hospitalized in the mixed medical-surgical unit of a university-affiliated medical center. The study was approved by the local Bioethics Committee (PCN/0022/KB/2/20). The article has been prepared in conformity with the Transparent Reporting of Evaluations with Nonrandomized Designs (TREND) guidelines.

### 2.1. Study Group

Patient recruitment started on 1 June 2020. All adult mechanically ventilated pa-tients admitted to the ICU who required radial cannulation for continuous, invasive blood pressure (IABP) measurement and who were undergoing haemodynamic monitoring with LidcoRapid were included in the study. There were no other additional inclusion criteria for the study. The exact reasons for the implementation of hemodynamic monitoring in the patients included in the study and the therapeutic decisions made on the basis of the obtained results were not analyzed. Exclusion criteria included: (1) spontaneous breathing, (2) prone position, (3) non-radial artery cannulation, (4) aortic regurgitation, (5) heart arrhythmia, (6) heart rate > 100/min, (7) intra-aortic balloon pump, (8) peripheral vascular disease, and (9) body weight < 40 kg.

### 2.2. Measurement Methodology

The arterial pressure curve was obtained by cannulating the radial artery with or without ultrasound control, depending on the operator’s preferences, using a BD Floswitch™ (Becton Dickinson, Franklin Lake, NJ, USA) 20 G cannula connected to a BeneView T8 monitor (Mindray, Shenzhen, China) using a set with a Transpac IT transducer (Icumedical, San Clemente, CA, USA). Each time, the proper position of the pressure transducer was verified (approximately 5 cm back from the sternum angle). The measuring line was flushed, and the monitoring set was automatically calibrated to generate an optimal pulse waveform (IABP) recording with a clearly visible dicrotic notch. The cardiac monitor screen settings were configured to display the IABP waveform recording at 25 mm s^−1^, with the greatest possible amplitude and the highest possible number of evolutions displayed simultaneously.

**Figure 1:**
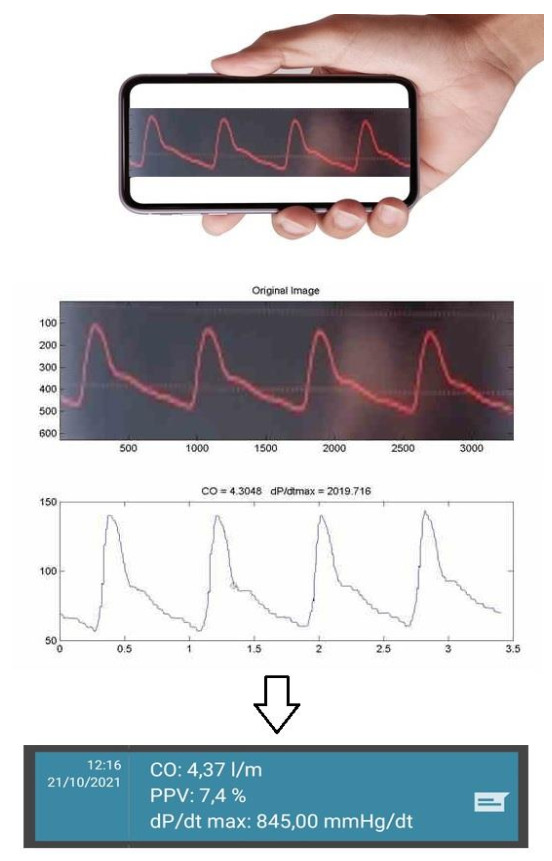
The method of measuring cardiac output by Capstesia.

The commercial Capstesia™ application (Galenic App, Vitoria-Gasteiz, Spain) was installed on a Huawei Nova 5T™ smartphone (Huawei Technologies Co., Ltd., Shenzhen, China) with the Android™ software and a 48 Mpx main camera. Switching on the CS application activates the camera, which takes a picture of the arterial pressure curve displayed on the monitor next to the patient’s bed. After taking the snapshot, the application asks for the SBP, DBP, and HR values at the time of taking the photo. After entering the data, the application shows the CO value. Completing the data with the CVP value results in automatic SVR calculations, similar to LR (Figure 1).

An external LR device, based on the manufacturer’s proprietary PulseCO™ (LiDCO Limited, Cambridge, UK) [14] algorithm, was connected to the output socket of the IABP monitor module. LR uses normograms to estimate vascular stiffness, which requires the user to enter basic demographic and anthropometric data: age, height, and weight. The LR then estimates the stroke volume (SV) and calculates the CO as the product of SV and HR. After entering a central venous pressure (CVP) value, the LR also automatically calculates SVR. The CO value (and the cardiac index—CI) is displayed continuously in real time, and the remaining parameters are displayed on the LR screen at minimum intervals of 5 min or more, as selected by the user. After the enrolment of the patient ensuring relative stabilization of macrocirculation parameters, one of the researchers took a picture of the IABP curve. Simultaneously, the other researcher noted the current CO-LR value without informing the CS researcher about the result. The first pair of obtained results was subjected to a comparative analysis of two methods (Bland–Altman analysis). Then, another 4 pairs of measurements were made (a total of 5 pairs of measurements for each patient) to determine the reproducibility of the CO-CS and CO-LR results. When the CS displayed the message that the measurement could not be performed, subsequent measurements were taken to obtain 4 pairs of measurements. Measurements were made in the same manner for all patients at similar time intervals (+/−60 s). Subsequently, two independent investigators retrospectively assessed the quality of the pulse waveform for the presence of a dicrotic notch. Consensus by both investigators was required to conclude that a dicrotic notch was present.

### 2.3. Statistical Analysis

Statistical analysis was performed using the procedures available in the licensed MedCalc Statistical Software version 18.2.1 (MedCalc Software bvba, Ostend, Belgium; http://www.medcalc.org (accessed on 10 December 2021); 2018) and SPSS version 22.0 (IBM Corporation, Armonk, NY, USA). The nature of the distribution of the variables was verified with the d’Agostino–Pearson test. Quantitative variables were presented in the form of median and interquartile range (IQR) or arithmetic mean and standard deviation. Qualitative variables were presented in the form of absolute value and percentage. Correlations between CO-LR and CO-CS were assessed using the Pearson linear correlation coefficient (r). The mean differences between the CO-LR and CO-CS values are presented in the Bland–Altman (B-A) diagram, taking into account the upper and lower limits of agreement (LOA). The percentage error was calculated from the formula: upper LOA-lower LOA/[mean CO-LR + mean CO-CS]/2] × 100%. Differences between the group of patients with the current dicrotic indentation and without a visible dicrotic indentation were assessed by Fisher’s exact test (qualitative variables) or the Kruskal–Wallis test (quantitative variables).

The criterion of statistical significance was *p* < 0.05.

### 2.4. Conflict of Interest

None of the researchers reported a conflict of interest in relation to the conducted study. The license to use the CS application was purchased with the researcher’s own funds (MPP). The LR device was the standard equipment used in the department where the study was conducted.

## 3. Results

The study group consisted of 16 patients (9 women and 7 men) with a median age of 66 (IQR 60–75). The characteristics of the demographic and clinical data selected are presented in Table 1.

A total of 80 pairs of measurements were made. The intra-class correlation (CCC) for CO-CS was 0.98 (95% CI 0.95–0.99. The median (IQR) CO-CS and CO-LR were 4.6 (3.9–5.7) and 5.5 (4.6–7.4) L min^−1^, respectively.

**Figure 2:**
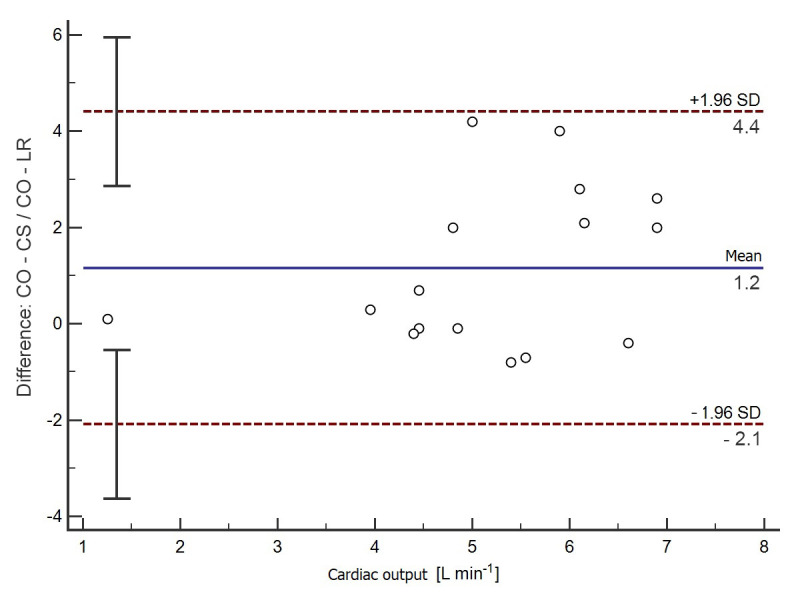
Bland–Altman analysis for the calculated CO-LR and CO-CS values. The solid horizontal line represents the mean value difference between the methods tested. The upper dashed line shows the upper compliance limit value (+1.96 SD). The lower dashed line shows the value of the lower compliance limit (−1.96 SD); SD—standard deviation.

In the B-A analysis, the CO-CS values were on average 1.2 L min^−1^ lower (95% CI from −2.1–4.4) than the CO-LR values (Figure 2), and the correlation between CO-CS and CO-LR was moderate (r = 0.5; 95% CI 0.01–0.8; *p* = 0.04).

**Figure 3:**
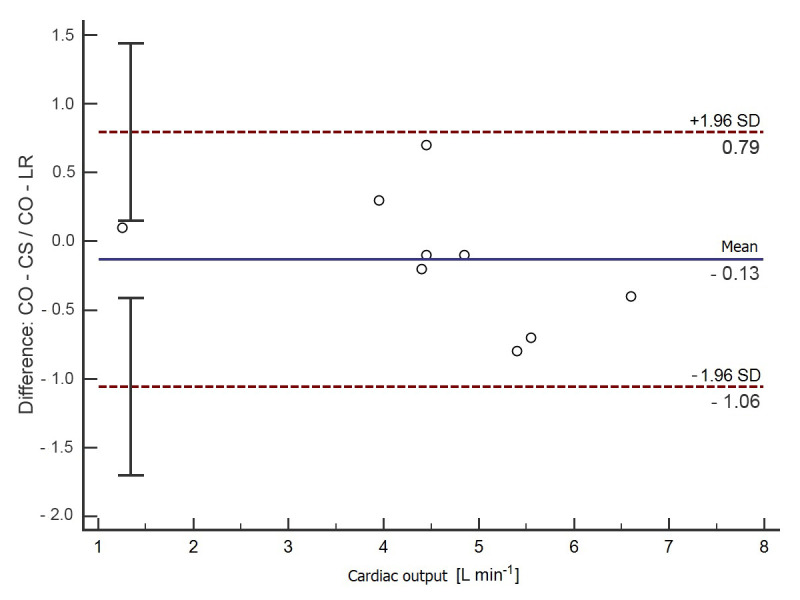
Bland–Altman analysis for the calculated CO-LR and CO-CS values in the group of patients with a visible dicrotic notch on the pulse wave curve (*n* = 9). The solid horizontal line represents the mean value difference between the methods tested. The upper dashed line shows the upper compliance limit value (+1.96 SD). The lower dashed line shows the value of the lower compliance limit (−1.96 SD); SD—standard deviation.

**Figure 4:**
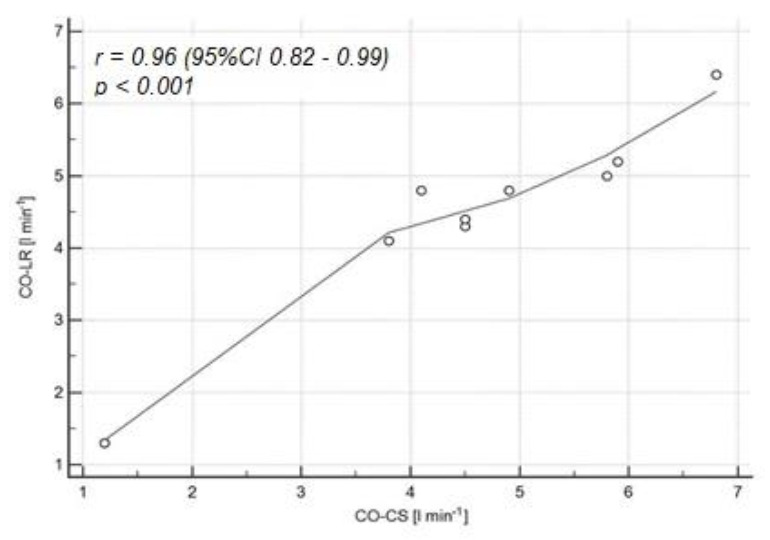
Correlation between the CO-LR and CO-CS values for the recording of the pulse wave curve with a visible dicrotic notch (*n* = 9). The solid horizontal line represents the mean value difference between the methods tested. The upper dashed line shows the upper compliance limit value (+1.96 SD). The lower dashed line shows the value of the lower compliance limit (−1.96 SD); SD—standard deviation.

After adjusting the results for the presence of the dicrotic notch in the pulse waveform in the group of nine patients with a visible dicrotic notch, the CO-CS and CO-LR values differed by only 0.1 L min^−1^ (95% CI −0.8–1.1) (Figure 3) in the B-A analysis, and the correlation between CO-CS and CO-LR was close to complete (*r* = 0.96; 95% CI 0.82–0.99; *p* < 0.001) (Figure 4). The characteristics of the clinical and demographic data selected depending on the presence of a dicrotic notch are presented in Table 2.

The percentage error of the measurements was 40% in patients with an identifiable dicrotic wave in the pulse waveform, compared with the percentage error of 125% for the entire study group.

## 4. Discussion

The aim of this study was to compare the CO values estimated by two hemodynamic monitors based on APCO technology in patients hospitalized in a mixed medical-surgical ICU. Although a large percentage error between CO-CS and CO-LR was observed in the entire study group, the results were comparable in the selected patients with a visible dicrotic notch on the pulse waveform. Peyton et al. [15] noticed that although most authors adopt a percentage error of <30% as a criterion of acceptability in relation to the reference method in clinical practice, it is only one of many factors determining the usefulness of the method tested. It is more important to follow the trend of changes than to rely on single, static values of hemodynamic parameters or to assess their accuracy. In our study, the intra-class correlation coefficient confirmed the very good repeatability of the measurements, although they took place in short time intervals and therefore with relatively stable macrocirculation parameters. Earlier, good agreement of the obtained results was confirmed between the selected minimally invasive hemodynamic monitors and CS under simulation and in a clinical environment [11,16]. A study by Santiago-Lopez et al. [17] showed significant agreement between CO values obtained with the CS and the Vigileo monitor (Edwards, Irvine, CA, USA) in 30 patients undergoing cardiac surgery. Shah et al. [18] also showed that CS may be an alternative method of hemodynamic monitoring to the Vigileo monitor in patients undergoing major oncology procedures. In 95% of the cases there would be a maximum discrepancy of 1.4 L min^−2^ m^−2^ between the CO measured by both devices. However, there are also other reports in which the compliance of CO-CS with CO measurements made, among other, the transpulmonary thermodilution method unacceptable, and the percentage error reached over 60% [13]. Although our study focused only on the comparison of CO-CS and CO-LR values, it is worth noting that there were successful attempts to use CS to assess fluid compliance using the PPV (pulse pressure variation) parameter. In the studies by Desebbe et al. and Joosten et al., the CS was compared to the EV1000 hemodynamic platform (Edwards, Irvine, CA, USA) with the FloTrack transducer (Edwards, Irvine, CA, USA) in patients undergoing major abdominal surgery. Intraoperative management of fluid therapy based on PPV values would result in completely opposite clinical decisions in only 1% of all measurements [12,19]. A recent study of the use of hypertonic saline in patients undergoing gastrointestinal surgery was the first prospective trial to use CS to make real-time clinical decisions and then to evaluate their effect. It has been shown that conducting GDT based on hemodynamic measurements with CS application reduced the amount of intraoperatively administered crystalloids and resulted in a favorable reduction in the positive fluid balance [11].

Finding a simple but reliable method of monitoring CO is driven by the need to make targeted therapeutic decisions without undue delay, based on available and validated hemodynamic algorithms. GDT is a well-established decision-making strategy for hemodynamic management in the ICU and operating theaters. GDT cannot be based on standard hemodynamic parameters such as HR, arterial BP, or CVP. Methods of advanced hemodynamic monitoring have to be employed, and dynamic parameters of fluid responsiveness should be used. In mechanically ventilated patients, minimally invasive self-calibrating techniques evaluating the stroke volume index based on pulse contour wave analysis proved useful in the context of the GDT [20]. Clinical decisions are usually made based on several parameters. CS fits perfectly into this scenario as an additional method of clinical assessment. Identification of patients who will not benefit from fluid therapy avoids harmful fluid overload, and after hemodynamic stabilization it enables the controlled correction of the cumulative positive balance with the use of diuretics and continuous renal replacement therapies [21]. Contrary to thermodilution, the CS application, based on the APCO method, does not provide several useful parameters, such as the extravascular lung water index (EVLWI), the intrathoracic blood volume index (ITBVI), and the global end-diastolic volume index (GEDVI) [22]. However, in the absence of thermodilution methods, repeated SV and/or PPV assessment in the CS application during the fluid challenge or passive test raising (PLR) may be a valuable supplement to the assessment of fluid susceptibility [23].

Our study was the first attempt to compare CS to the LidcoRapid hemodynamic monitor, as well as the first such study conducted in the ICU patient population. It provided new data, although it was subjected to the risk of error because of the low number of patients recruited due to the need to meet stringent APCO evaluation criteria. Other limitations were consistent with the previous reports of other researchers and included: (1) the risk of parallax error; (2) reflecting the monitor image in unfavorable lighting, resulting in a poor-quality IABP recording; and (3) the need to verify the correct processing of the curve image by the CS application before making a clinical decision. Nevertheless, in our opinion, one of the most serious limitations of CS was the occurrence of frequent messages about the inability to perform the measurement despite the good quality of the snapshot and access to a fast internet connection, which, combined with the need to edit the obtained snapshot each time and enter the SBP, DBP and HR values, greatly extended the time to obtain the result. This could result in a potentially dangerous situation where the operator concentrated on taking multiple pictures of the pulse wave at the expense of observing the patient’s clinical condition. This significantly limits the attempt to safely use a CS in a situation of rapid changes in a patient’s clinical condition, unless additional personnel are involved. Nevertheless, this relatively new mobile health technology could potentially benefit patients who should be treated as suffering from a medical emergency, namely patients with sepsis or septic shock. According to the guidelines, these patients should have invasive arterial monitoring instituted as soon as logistically possible [24]. Having invasive arterial monitoring in place, these patients would benefit from early hemodynamic assessment with this new software.

## 5. Conclusions

The Capstesia^TM^ smartphone software may constitute an alternative method for CO assessment in patients meeting APCO evaluation criteria with a clearly discernible dicrotic notch on the arterial pulse pressure waveform. It is necessary to confirm the observations obtained on a larger group of patients. It seems necessary to improve the technology in patients with a non-discernible dicrotic notch on the arterial pressure waveform.

## Figures and Tables

**Table 1:** The characteristics of the demographic and clinical data selected.

Variable	Value
Female (*n*, %)	9 (56%)
Age (years)	66 (60–70)
Height (m)	1.65 (1.60–1.75)
Body weight (kg)	70.5 (67.5–80)
BMI (kg m^−2^)	26 (24–28)
BSA (m^2^)	1.83 (1.67–1.96)
Heart Rate (1 min^−1^)	77 (72–93)
Systolic blood pressure (mmHg)	120 (102–134)
Diastolic blood pressure (mmHg)	58 (52–67)
Mean Arterial Pressure (mmHg)	76 (71–91)
Pharmacological support of the cardiovascular system	
Norepinephrine, *n* (%) Dose (ug kg^−1^ min^−1^)	10 (63%) 0.1 (0.1–0.2)
Epinephrine, *n* (%)	2 (13%)
Dose (ug kg^−1^ min^−1^)	0.13 (0.05–0.2)
Argipressin, *n* (%)	1 (6%)
Dose (units min^−1^)	0.02
Main diagnosis	
Septic shock (*n*, %)	8 (50%)
Hypovolemic shock (*n*, %)	1 (6%)
Subarachnoid hemorrhage (*n*, %)	5 (32%)
ARDS (*n*, %)	2 (12%)

Qualitative variables are presented as absolute value and percentage; quantitative variables are presented as median and interquartile range (IQR); BMI—body mass index; BSA—body surface area; ARDS—acute respiratory distress syndrome.

**Table 2:** Characteristics of selected demographic and clinical data depending on the presence of a dicrotic notch.

Variable	Value		*p*
	Dicrotic Notch (+)	Dicrotic Notch (−)	
*n* (%)	9 (56%)	7 (44%)	0.6
Age (years)	66 (62–73)	64 (60–76)	0.7
Height (m)	1.65 (1.60–1.75)	1.75 (1.61–1.79)	0.4
Body weight (kg)	70 (68–76)	80 (66–88)	0.4
BMI (kg m^−2^)	25.7 (22.0–28.2)	25.7 (24.9–28.1)	0.7
BSA (m^2^)	1.77 (1.66–1.90)	1.96 (1.70–2.05)	0.2
Heart Rate (1 min^−1^)	74 (68–83)	92 (77–94)	0.3
Systolic blood pressure (mmHg)	120 (100–133)	120 (106–134)	0.9
Diastolic blood pressure (mmHg)	66 (55–84)	52 (48–60)	0.1
Mean Arterial Pressure (mmHg)	85 (73–99)	72 (68–85)	0.2
Pharmacological support of the cardiovascular system			
Noradrenaline, *n* (%) Dose (ug kg^−1^ min^−1^)	5 (31%) 0.1 (0.08–0.2)	5 (31%) 0.1 (0.2–0.3)	0.5 0.9
Adrenaline, *n* (%)	2 (13%)	-	-
Dose (ug kg^−1^ min^−1^)	0.13 (0.05–0.2)	-	-
Argipressin, *n* (%)	1 (6%)	-	-
Dose (units min^−1^)	0.02	-	-

Qualitative variables are presented as absolute value and percentage, quantitative variables are presented as median and interquartile range (IQR); BMI—body mass index; BSA—body surface area.

## Data Availability

Data are available from the authors of the study.
